# Recommendations for Improving Systemic Lupus Erythematosus Care From Black Adults: A Qualitative Study

**DOI:** 10.1001/jamanetworkopen.2023.40688

**Published:** 2023-10-31

**Authors:** Bhaavna Yalavarthi, Johari Summerville, Nikki Farahani, Lillian Z. Xiao, Christine Yu, Deena Aboul-Hassan, Sia Rajgarhia, Daniel J. Clauw, J. Michelle Kahlenberg, Melissa DeJonckheere, Rachel S. Bergmans

**Affiliations:** 1 Medical School, Department of Anesthesiology, University of Michigan, Ann Arbor; 2 Medical School, Department of Internal Medicine, University of Michigan, Ann Arbor; 3 Medical School, Department of Family Medicine, University of Michigan, Ann Arbor

## Abstract

**Importance:**

Racial inequities in incidence, morbidity, and mortality are a defining feature of systemic lupus erythematosus (SLE). Health care systems are integral to addressing these inequities. However, qualitative evidence that highlights Black SLE care experiences is limited.

**Objective:**

To identify opportunities for improving SLE care based on the experiences and perspectives of Black adults with SLE.

**Design, Setting, and Participants:**

In this qualitative study, an interpretive description approach was used and data were analyzed using inductive thematic analysis. Semistructured interviews with Black adults in Michigan who were diagnosed with SLE were conducted. Interviews occurred from November 2, 2021, to July 19, 2022, and data analysis occurred from May 6, 2022, to April 12, 2023.

**Main Outcomes and Measures:**

Deidentified transcripts from the interviews were analyzed to develop themes that focused on opportunities to improve quality of care and symptom management.

**Results:**

The participants included 30 Black adults with SLE (97% women; mean age, 41 years; range, 18-65 years). Four main themes were identified: (1) awareness of SLE signs and symptoms before diagnosis (participants emphasized delays in diagnosis and how knowledge concerning SLE could be limited in their families and communities); (2) patient-clinician interactions (participants faced discrimination in health care settings and talked about the value of coordinated and supportive health care teams); (3) medication adherence and health effects (participants experienced a range of adverse effects from medications that treat SLE and described how monitoring medication use and efficacy could inform tailored care approaches); and (4) comprehensive care plans after diagnosis (participants reported persistent pain and other symptoms despite treatment). In the context of disease management, participants emphasized the importance of behavioral change and the negative impact of social risk factors.

**Conclusions and Relevance:**

The findings of this qualitative study suggest how limited information about SLE, experiences of racism, treatment regimens, and social risk factors may affect Black people with SLE. Future research should further engage and include Black communities within the context of treatment and intervention development to reduce racial inequities.

## Key Points

**Question:**

What opportunities are there for improving systemic lupus erythematosus (SLE) care, based on the experiences and perspectives of Black adults with SLE?

**Findings:**

In this qualitative study including 30 Black adults with SLE, 4 main themes influencing quality of care and symptom management were identified. These themes included (1) awareness of SLE signs and symptoms before diagnosis, (2) clinician-patient interactions, (3) medication adherence and health effects, and (4) comprehensive care plans after diagnosis.

**Meaning:**

The findings of this study suggest how experiences of racism, limited information about lupus, treatment regimens, and social risk factors may affect Black adults with SLE.

## Teaser

This qualitative study presents the results of interviews with Black individuals with systemic lupus erythematosus on their experiences and perspectives regarding health care.

## Introduction

Systemic lupus erythematosus (SLE) is a chronic autoimmune condition with no cure that can cause a range of symptoms including skin inflammation, severe fatigue, widespread pain, and kidney disease.^1^ As seen in many chronic conditions, racial inequities are also a defining feature of SLE. The incidence of SLE is 3 to 4 times higher among Black adults compared with White adults,^2^ and mortality due to SLE is 2 to 4 times higher among Black patients than White patients.^3^ The factors associated with these health inequities are many and likely include the influence of systemic racism on where people live and environmental exposures, which are implicated in SLE etiology,^4^ as well as inequities related to health care quality. For example, Black people are nearly twice as likely to report discrimination in health care settings compared with White people.^5^ The underrepresentation of Black people as participants in SLE research^6^ is also a concern because it limits the ability to ensure treatment efficacy and develop care approaches that are sensitive to the needs and preferences of these individuals.

Qualitative research may help improve the quality of care that people receive through providing a detailed and nuanced picture of their experiences.^7^ For example, qualitative research in rheumatoid arthritis revealed that ideal care approaches may depend on disease duration, and patients want treatment plans that value their overall wellness.^8^ However, qualitative evidence that highlights Black SLE care experiences is limited.

In this study, we aimed to identify opportunities for improving care based on the experiences and perspectives of Black adults with SLE. Using a spotlight approach provides the opportunity to address inequities by developing SLE care recommendations that prioritize the perspectives of Black people, who are known to experience social disadvantages and be underrepresented in research. This aligns with the principles of community-engaged research and health equity because it centers Black voices in ways that are not possible within comparative studies.^9^

## Methods

### Study Design

We used an interpretive description design, which originated in nursing science and allowed us to identify opportunities to improve clinical care by drawing from participant experiences.^10^ Data came from one-on-one semistructured interviews with Black adults with a diagnosis of SLE. The University of Michigan approved this study and designated it exempt from ongoing institutional review board review. Thus, obtaining written informed consent was not required. However, we provided individuals with a copy of the informed consent document for review with study staff via video or teleconference prior to enrollment. Participants received $25 for completing the study interview. We followed the Standards for Reporting Qualitative Research (SRQR) reporting guideline.^11^

### Reflexivity

Reflexivity is critical for reducing bias in qualitative data collection and analysis.^12^ Three preconceptions informed our work: (1) SLE is more common and more severe among Black adults than White adults,^2,3,13^ (2) the unequal and unjust distribution of opportunities and resources due to public policies and social norms contributes to health inequities,^14^ and (3) symptoms such as pain and fatigue may not be adequately addressed due to a lack of objective measures; instead, SLE treatment guidelines emphasize inflammation and immunologic markers.^15^ Our study team included a community-engaged researcher (R.S.B.) and a clinician (J.M.K.) who interacted with members of the study population in other settings (eg, lupus awareness and education events, SLE treatment, and clinical research).

### Data Collection

Eligible participants were adults, self-identified as Black or African American, resided in Michigan, and reported an SLE diagnosis. We recruited a convenience sample from October 26, 2021, to July 19, 2022, by posting study flyers in public places, advertising on social media, sharing information about this study with lupus advocacy organizations, and through a registry of people with SLE at an academic medical center. Interviews occurred from November 2, 2021, to July 19, 2022, and data analysis occurred from May 6, 2022, to April 12, 2023. We determined eligibility using a close-ended survey (eAppendix 1 in Supplement 1).

Authors with expertise in health equity (R.S.B.), qualitative research (M.D. and R.S.B.), and rheumatology (D.J.C. and J.M.K.) guided the creation of a comprehensive interview guide with input from people who have SLE or know someone with SLE that included close-ended survey questions about social risk factors and symptom management (eAppendix 2 in Supplement 1). We conducted semistructured interviews remotely, using video and teleconference software compliant with the Health Insurance and Portability and Accountability Act. Interview length ranged from 23 to 153 minutes. We transcribed and deidentified the interviews before analysis. We completed 30 interviews total and reached data saturation after 18 interviews, which we determined by monitoring the scope of responses during data collection.

### Data Analysis

We used an inductive thematic analysis approach, summarized in the Figure. First, we extracted demographic, social, and health-related characteristics to describe the study sample. We then open-coded the transcripts to identify salient concepts from participants’ words, and we created profiles for each participant, including memos and conceptual diagrams (eFigure in Supplement 1), to represent the relationship of concepts within participants.^16^ Next, we used the within-person profiles to create categories that characterized the data across participants. During weekly meetings, our analysis team reviewed and discussed any analytical discrepancies that arose during category creation until we reached consensus.

**Figure.**
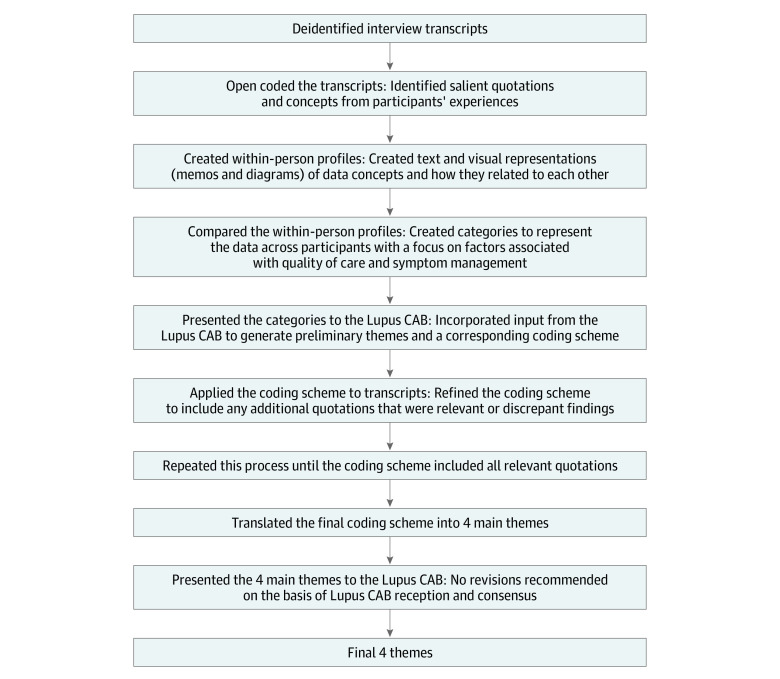
Analysis of the Deidentified Interview Transcripts to Develop the Main Themes Lupus CAB indicates University of Michigan Community Advisory Board for Lupus Care and Research.

After developing a set of categories for the factors that influenced quality of care and symptom management, we presented these preliminary categories to the University of Michigan Community Advisory Board for Lupus Care and Research (Lupus CAB) on November 9, 2022. The Lupus CAB included Black women with SLE and other representatives of Black communities in Michigan, which allowed our category development to reflect input from the study population and increased the trustworthiness and rigor of our research.^17,18^ From these categories, we developed preliminary themes. To refine these preliminary themes, we generated a corresponding coding scheme and applied it to the transcripts using MAXQDA 2022 (VERBI Software). As an iterative process, we refined our themes to include any findings that conflicted with the initial coding scheme. This resulted in the final 4 main themes that we confirmed with the Lupus CAB in May 2023.

## Results

This study included 30 Black adults (97% women; mean age, 41 years; range, 18-65 years) (Table 1). Among participants, 40% reported financial hardship, including food insecurity and skipping health care services to save money. All but one participant experienced fatigue. Nearly all participants reported depressive and/or anxiety symptoms (93%), and 70% reported multisite chronic pain. Participants’ first signs and symptoms of SLE before the diagnosis were headaches, muscle aches, fatigue, rash, hair loss, joint pain and swelling, light sensitivity, dizziness, lightheadedness, fever, blood clots, shortness of breath, and loss of appetite.

**Table 1.** Sample Description of Black Adults in Michigan With SLE

Participant ID	Age range, y^a^	Marital status	Financial hardship^b^	Delays in diagnosis
1	55-59	Single	Yes	No
2	50-55	Divorced	No	No
3	20-24	Single	No	No
4	30-34	Single	Yes	Yes
5	40-44	Single	No	No
6	60-64	Divorced	No	Yes
7	25-29	Married	No	No
8	25-29	Married	Yes	No
9	15-19	Single	No	No
10	20-24	Married	No	No
11	45-49	Single	No	Yes
12	25-29	Single	Yes	No
13	40-44	Single	Yes	No
14	45-49	Married	No	Yes
15	65-69	Widowed	No	No
16	40-44	Single	Yes	Yes
17	30-34	Married	No	No
18	55-59	Single	Yes	No
19	60-64	Divorced	No	No
20	45-49	Married	No	Yes
21	50-54	Single	Yes	Yes
22	40-44	Married	No	Yes
23	25-29	Domestic partner	No	No
24	30-34	Married	No	Yes
25	40-44	Married	No	No
26	45-49	Divorced	Yes	Yes
27	45-49	Married	No	Yes
28	40-44	Single	Yes	No
29	30-34	Married	Yes	No
30	35-39	Domestic partner	Yes	Yes

Abbreviation: SLE, systemic lupus erythematosus.

^a^ Age range provided to protect participant identities.

^b^ Refers to food insecurity, housing insecurity, lack of access to transportation, and/or skipping medication and physicians’ visits to save money.

We developed 4 main themes that reflect opportunities to improve SLE care (Table 2). Each theme includes examples with additional detail about participants’ qualitative experiences.

**Table 2.** Four Main Themes That Describe Opportunities to Improve SLE Care

Main themes and examples of qualitative experience	Quotations from participant experience^a^
**Awareness of SLE signs and symptoms before diagnosis**	
Receiving and seeking appropriate and timely care	My experience being diagnosed with lupus was pretty hectic. I actually started to exhibit lupus symptoms years before my diagnosis. (P4)
	Those [full body ache] episodes happened probably once a month… My mom did take me to the ER one time because it was so bad, and they gave me a shot in my butt, and the pain went away. And that was it, it was no further testing. It was 7 y, 6 or 7 y [between my initial symptoms and my SLE diagnosis]. (P14)
	I, thought I had the flu. My aunt thought I had the flu, but lupus was never a topic in my family. So, it was never something that we thought that I had. (P16)
	I remember telling my best friend … this is one of the worst things I’m ever going to say, and I feel so guilty about it, but I wish I had cancer … I wish it was something definitive like that because cancer would be easier than not knowing. (P24)
Family history	This is another thing that made my case harder. I was adopted when I was younger, and since being diagnosed I found out who my biological mother is... I never got to meet her since she passed a few years ago and my biological father, he was killed, he was hit by a car I think in 2005. So I don’t really have any sort of familial history or any sources that would be concrete and like absolute. (P25)
	No, I wasn’t surprised [by my SLE diagnosis] just because my other family members had it. (P30)
**Patient-clinician interactions**	
Exposure to racism and implicit bias	I’m not the person to say it’s because I’m Black. But I think it is legit... I’m not a priority [to health care professionals] … Their actions are telling me they don’t really care. They don’t really care. (P16)
	There are some systematic things when it comes to not just people of color but even women of color. Pain levels aren’t taken seriously. “We’re faking.” I remember one rheumatologist I saw, he said, “What’s your pain level?” I said, “It’s a 10 and if it could be plus, it would be plus.” And he’s kind of joking, “No really, what’s your pain?” I stopped him and I said, “I gave birth to two boys, they didn’t hurt. This hurts.” I don’t want to always have to say, “Do you have a doctor of color in your office?” for me to feel like I’m going to be heard, seen, or paid attention to. (P20)
	It was really hard for my symptoms to be taken seriously, for physicians to take a deep dive into and to not exhibit any bias or assuming that I’m just looking for opiates and other pain management. Don’t get me wrong, I had a lot of pain, but I really wanted to get down to the cause and not just manage the symptoms. [The physicians] probably could have checked their bias at the door. And not just about my race, but about my gender and about my symptoms in general. They could have listened to me and I’ve moved past the point of grief of like just being angry and like I’m going to sue the doctor for malpractice, you know. I was so angry, but I think being African American, and even before I opened my mouth, presenting as a person of color and as presenting as a woman. (P24)
Compassionate, communicative, and invested clinicians	[My doctor] was great. She explained to me about the condition, she explained that it is manageable, and that we were going to be partners and finding different methods to get through this. She asked me what my goals were, and I told her what they were, and then we’ve worked on that plan. (P2)
	The advice that I would give [to other people with SLE] is, number one, make sure that you find a good doctor where you feel seen and heard. If you feel as though you’re being gaslighted, if they’re telling you it’s all in your head, find another doctor because your health is much too precious for you to continue on with treatment with a physician where you don’t feel seen, you don’t feel heard. (P5)
	I do like that [my doctor] actually listens to me first and tries to connect with me. She understands what I’m saying. (P9)
	When they told me I was going to have to apply for disability, um, I would like to have known why. I didn’t know that this disease makes you tired, I didn’t know that it was going to make me incapable of doing the job that I did. And I would have liked to know why, instead of just being told you need to stop working. (P18)
	I do know how to remove myself from one doctor and go... not to somebody that says yes, but to someone who understands the importance of that partnership. (P20)
Care coordination	My pharmacist knows what I’m allergic to… The pharmacy was the one that identified one of the medications that I don’t need. [The pharmacist said], “I think the dose is just too high. I’m kind of concerned,” and so he called the doctor, and she changed the dosage. (P2)
	Because most of the things that go on with me are lupus or lupus related, it’s really my rheumatologist, and it’s also my nephrologist. So those are the two main doctors as far as my treatment goes. The good thing about that too is that, because they’re in the same office, they’re literally like right down the hall from each other, then they’re able to communicate with each other as far as my care. (P5)
	All [my doctors] are all willing to work in tandem together, which is exceptional. They’re willing to have doctor to doctor … discussions, which is fantastic. (P24)
**Medication adherence and health effects**	
Medications can be cost-prohibitive	My medication that was working best for me was covered by my insurance. Because one of the ingredients was not approved by the FDA, they declined the whole thing. So, I would have to pay out of pocket in order to feel good. (P28)
	One of the medications, I do have to pay for it. It’s $110 or something a month. So, me not working right now, and I’m just going to school. That’s hard. (P30)
Medication complications	I figured it out that they had to take me off [Plaquenil] because the side effect is blindness. And so I will suspect the glaucoma and my astigmatism in my cataracts advanced real fast. (P15)
	If my symptoms aren’t really, really bad, I’ll skip a dose or two, which my doctor doesn’t like, but I have trouble just thinking, damn, I’m taking out of all this medicine. Like, what is it doing? Is it going to help in the long run? Or is it going to hurt me more in the long run? (P30)
Tracking medication adherence and response	I would document what had happened a couple of days before and when I had my doctor visits, we would go over my log. And that process helped me to figure out the flares and [my doctor] also taught me how to adjust my meds, to go up or down. (P6)
	I had to let the doctors know so many times they’ve given me just, it was too much [medicine]… And thank God for this nurse. I was having an infusion, and she was like, “You shouldn’t be reacting this way. I’m going to have them adjust your medication.” (P11)
	[My doctor said], “You’re not going to run out [of medication]. If I say do something, let’s say, try something try half a dose, we’re going to do that. And if you have any trouble with side effects, let me know. We will treat you for side effects.” (P29)
**Comprehensive care plans after diagnosis**	
Self-directed lifestyle and coping approaches for symptom management	I will forget things, so I make myself notes or have like these sticky pad things all over the place to remember. (P4)
	I’m doing exercise, eating healthy, and not smoking so that’s helping me. (P7)
Integrative health care	I wish there was something that I could do besides taking narcotic pain medicine for the pain. I wish it was something else that I could do. It’s very painful and it’s something I’m going to have to eventually come to terms with. (P29)
	Being depressed about being sick, that’s the biggest challenge that most people with lupus have. Because they know that it’s almost a death sentence. When you’re diagnosed with lupus, you know it’s going to attack your heart, your brain, your lungs, or your kidneys. So, the more support that [people with lupus] can get from not even a psychiatrist, but a maybe a therapist or psychologist, somebody that can talk to them and not just listen and not say anything, they need to talk back to the person. Telling them, look, “This is what you need to do,” and they should be knowledgeable on the disease itself to let them know that even though you have it, don’t give up. Keep going. Keep moving. Keep doing things. Keep having fun. Do things that you never thought you can do. Just go and do it. (P19)
The influence of social factors on care	I hired this nutritionist, which I don’t have her now because the economy and she’s way too expensive. (P4)
	The only way that I’m confident now about my food sources is because I’m on supplemental income. I get the food card. But before then, I remember only getting $16 a month and that was very difficult. With this new administration in the pandemic a lot has changed. But I did have food insecurity for a long time when I first got diagnosed. (P18)
	I also started therapy within the last month because I found out that my insurance will actually cover it. So that’s been helpful. But I’ve never been in therapy before. (P28)

Abbreviations: ER, emergency room; FDA, US Food and Drug Administration; SLE, systemic lupus erythematosus.

^a^ Quotations from participant experiences are given intact.

### Theme 1: Awareness of SLE Signs and Symptoms Before Diagnosis

As participants explained the process of receiving an SLE diagnosis, some people experienced delays in diagnosis and emphasized limited knowledge concerning SLE in their families and communities. The nature of initial SLE symptoms could affect the diagnosis timeline. For some, classic SLE symptoms, such as the butterfly rash, increased the speed of diagnosis. For others, having pain and fatigue as the first signs of SLE contributed to an initial misdiagnosis. At times, diagnosis was the result of a hospitalization or multiple hospitalizations. One participant told her physician that she thought she had lupus (“They just put it on my chart that I was ‘self-diagnosed lupus.’” Participant [P]28), but the diagnosis was not official until “a couple of years after that.”

Another factor that appeared to affect the speed of diagnosis included knowledge of family history regarding SLE. While several participants talked about having a family history of SLE, this information did not always facilitate a personal diagnosis. For example, one participant’s sister was diagnosed with SLE before her, but she did not find out until after her own diagnosis years later (“I found out [my sister] had lupus [2 years before my diagnosis]. She actually kept it from the family.” P22). Two participants talked about being adopted and could not draw on their biological family health histories to inform their care.

Another important feature of this diagnosis process was the level of awareness that participants and their health care professionals had concerning SLE signs and symptoms. Often the SLE symptom and diagnosis journey starts well before people are evaluated by a rheumatologist, and many participants did not receive appropriate or timely care. One participant who waited a long time to receive the diagnosis remembers wishing she had cancer to take away the burden of not knowing. Another participant specifically highlighted the need for more awareness of SLE symptoms within their community, saying, “Make it a little bit more well-known, bring more awareness about the disease itself. It will probably help.” (P26)

### Theme 2: Patient-Clinician Interactions

This theme was characterized by participants’ descriptions of discrimination in health care settings and the value of coordinated and supportive health care teams. Participants explained that their care was affected by how clinicians treated them. For example, participants experienced racism, were labeled as drug-seeking, and had their symptoms dismissed or disbelieved by clinicians (eg, “With me being an African American woman, I have experienced going to the ER and experiencing medical bias, that medical racism in the field.” P5). When participants did not have the relationship that they wanted with their physicians, they sought care elsewhere. One participant described how being stigmatized by physicians made her hesitant to advocate for herself: “You go back and forth with trying to be an advocate for yourself, without being too pushy. You don’t want them to view you as a complainer or a drug seeker, or [the doctor thinks] I want to [get] disability. I want to work and get myself back because disability income is still not going to be the lifestyle that I wanted to establish for myself and went to school for.” (P28)

Conversely, participants explained the positive impact of physicians who took the time to explain what was going on and develop a treatment plan. Participants also described how SLE requires care from several types of health care professionals, including dermatologists, pharmacists, nutritionists, psychotherapists, internists, nephrologists, and nurse practitioners in addition to rheumatologists, and they talked about how their care was better when their health care professionals worked “in tandem” (P24) and communicated with each other.

### Theme 3: Medication Adherence and Health Effects

Under this theme, participants shared how SLE medications come with challenges, such as adverse effects that affect medication adherence. For example, medications could be cost-prohibitive, especially when participants were unable to work due to SLE or when their insurance no longer covered certain prescriptions. An inability to obtain medications in the future due to cost or insurance-related restrictions was a fear for multiple participants, and for some, this altered their treatment plan (eg, “I’ve been [receiving] prednisone for 10 years. Well, 11 years. Which I’m not supposed to be [receiving] it that long. They were going to [prescribe] Benlysta, but my insurance wouldn’t cover it.” P1).

Participants described the adverse effects from receiving glucocorticoids and other prescription medications, including swelling, weight gain, osteonecrosis, glaucoma, nausea, and “going blind” (P16). At times, the adverse effects of medication reduced treatment adherence. Participants talked about skipping doses without telling their physicians or throwing medications away because of the adverse effects. Additionally, a trial-and-error approach to medication was confusing and frustrating for some people: “I can’t even explain to [the doctors] what’s been going on with me between now and the last 3 months and [they] just throw me [medications to try]. We’ll try this and see if this works. And then I’ll try the medication that caused my potassium to drop.” (P28) One participant had unique concerns about drug interactions due to taking medications for multiple conditions (“Because I have psoriasis and lupus some medications don’t work together … so, you’ve got to be really careful with the medications.” P27)

For other participants, talking about medication adherence and symptoms with health care professionals helped with tailoring their treatment plan. Despite adverse effects, achieving optimal health was a core motivation for taking prescription medications: “I don’t like medication but when I got my disease, I think it’s very essential for me to take medication because I love my body. I love my health. My health is my priority.” (P8)

### Theme 4: Comprehensive Care Plans After Diagnosis

In this theme, participants reported that they would benefit from comprehensive care approaches that did not focus on a single dimension of their health. For example, participants used a range of lifestyle strategies to help manage SLE, including relaxation techniques, spirituality, sun protection, nutrition, and physical activity. Participants explained how SLE could require adopting new behaviors like changing their diet (eg, “I cut out red meat, so I don’t eat pork or beef anymore. And I try not to drink alcohol too much.” P3), giving themselves permission to rest (eg, “One of the things that I’ve done is allowing myself to rest a lot more, because beforehand that wasn’t something that I did.” P4), and finding ways to reduce the impact of their symptoms, like memory problems (eg, “My brain does not store information like yours might. I have to have references. I’m a sticky note girl.” P16).

Participants described wanting information about integrative, nonpharmaceutical treatments. A participant said, “We don’t want to take [just pain medicine and steroids] …. There has to be some other form of treatment that they have to come up with.” (P4). Participants also talked about how treating pain, fatigue, depression, and anxiety was especially important to their health. This included the value of psychotherapy (eg, “When they told me that I was going to die [after receiving my lupus diagnosis], if I would have let that be it, I would have stayed in that wheelchair and I would not have probably made it. So, water your brain. Seek out therapy.” P16) and educational resources for SLE management. For example, one participant was afraid after diagnosis because of the lack of information about SLE prognosis and management: “My first reaction was that I was very scared… There wasn’t any information out there on the internet. [There wasn’t] any place that I could go, or I could call to just gain more information about it. It was just, you have lupus and that was that.” (P5)

Participants elaborated how social risk factors can affect SLE management, including food insecurity, literacy, and neighborhood walkability. For example, while some participants managed their SLE with the help of self-guided research, this may not be an option for everyone. As one person explained, “A few years ago, we had a 52% functioning illiteracy rate [in my community]. So, you can’t minimize and assume that people can read and understand.” (P11). Another participant talked about how their insurance status affected their continuity of care and where they could go for health care: “I had to find another provider and I got a different insurer now. So having that type of insurance, most providers don’t want to take you on as the patient .… The providers are limited.” (P28)

Alternatively, the impact of SLE can be minimal for those with comprehensive social support. A participant described her experience: “I do say that coming from a point of privilege. I have a great job, my husband has a great job, I have amazing benefits. I’ve never had to worry about food insecurity, or spousal abuse, or having enough money to put gas in my car so I can go to work … I’ve been exceptionally lucky and blessed in that aspect. So yeah, lupus ironically has made me healthier, I think, which is weird to say, but yeah.” (P24)

## Discussion

Based on semistructured interviews with Black people in Michigan who were diagnosed with SLE, we identified 4 main themes that influenced quality of care and symptom management: awareness of SLE signs and symptoms before diagnosis, patient-clinician interactions, medication adherence and health effects, and comprehensive care plans after diagnosis. This aligns with prior work, including a study that used data from a large, online forum for people with SLE and related autoimmune diseases in which participants described the harms of a delayed diagnosis, having symptoms dismissed, a desire for invested health care professionals, and valuing coordinated and multidisciplinary care.^19^ Our study extends existing evidence by focusing on the perspectives of a marginalized patient population that faces inequities concerning SLE incidence, morbidity, and mortality. More specifically, our findings highlight how limited information about SLE, experiences of racism, treatment regimens, and social risk factors could be targeted to improve the quality of SLE care.

Consistent with our findings, reducing the time to diagnosis in Black populations could improve SLE prognosis and alleviate health inequities. For example, when lupus nephritis (a complication of SLE) is diagnosed before kidney insufficiency, this prevents kidney failure.^20^ Kidney damage can affect over half of recently diagnosed Black patients, at a rate that is 2 times higher relative to White patients.^13^ Increasing awareness of SLE and diagnostic criteria could be one approach to reducing delays, which is consistent with recommendations from the National Academy of Medicine for a range of difficult-to-diagnose conditions.^21^ Clinicians in primary care and emergency medicine may especially benefit from targeted educational campaigns about SLE. This is because primary care and emergency medicine professionals are likely a first point of contact for SLE, and misdiagnosis is more common among nonrheumatologists—partly due to a dependence on a positive antinuclear antibody test.^22^ Additionally, as our evidence suggests, family history for SLE may be unknown or incomplete, so it may not be a reliable resource when making decisions about diagnostic testing or referrals.

Emphasized within our data, efforts to reduce exposure to discrimination in health care are critical to improving the quality of care that Black people receive both before and after an SLE diagnosis. The expectation of experiencing discrimination can cause delays in care seeking.^23^ Additionally, racism and bias are inconsistent with the Hippocratic Oath and are known to prevent treatment adherence and increase the risk of morbidity and mortality.^24^

In this study, participants explained the need for more comprehensive treatment plans that sufficiently addressed all dimensions of their health. More detailed monitoring of medication adherence and symptoms over time may provide one solution. For example, glucocorticoids and biologics are the first-line of treatment for SLE inflammation and, while effective, these therapies come with many adverse effects that may prevent continued use.^25,26^ Additionally, most of the therapeutic interventions offered to people with SLE treat inflammation, which leaves noninflammatory sources of pain and other symptoms untreated and inadequately addressed.^27^ Thus, integrative, multicomponent care that uses a mix of pharmaceutical and nonpharmaceutical treatments may prove effective for SLE symptoms that do not respond to standard approaches.

### Limitations

There are limitations to this study. Black communities are not homogeneous, and our findings should not be generalized to all Black people with SLE. There are likely alternative and additional opportunities to better SLE care that could be identified within other populations, for example, from another state or region, or patient subgroups.

## Conclusions

In this qualitative study with Black adults, we drew from participant experiences to identify areas for improvement in SLE care, such as preventing exposure to racism, discrimination, and bias and ensuring that people receive additional treatment as needed for their symptoms. Future research should further engage and include Black communities within the context of SLE treatment and intervention development to reduce racial inequities in SLE care and research.

## Notes

Supplement 1. **eAppendix 1.** Eligibility Survey and Inclusion Criteria **eAppendix 2.** Semi-Structured Interview Guide for Assessing the Perspectives and Experiences of Black Adults With SLE **eFigure.** Example of a Conceptual Diagram From the Within-Person Profiles

Supplement 2.
Data Sharing Statement
