# Supplementary material for: Recommendations for Improving Systemic Lupus Erythematosus Care From Black Adults: A Qualitative Study
Source: JAMA Netw Open. 2023 Oct 31;6(10):e2340688. doi: 10.1001/jamanetworkopen.2023.40688 (PMC10618846; doi:10.1001/jamanetworkopen.2023.40688)
Supplement: Supplement 1. — eAppendix 1. Eligibility Survey and Inclusion Criteria eAppendix 2. Semi-Structured Interview Guide for Assessing the Perspectives and Experiences of Black Adults With SLE eFigure. Example of a Conceptual Diagram From the Within-Person Profiles [file jamanetwopen-e2340688-s001.pdf]

Supplementary Online Content

Yalavarthi B, Summerville J, Farahani N, et al. Recommendations for improving systemic lupus erythematosus care from Black adults: a qualitative study. *JAMA Netw Open*. 2023;6(10):e2340688. doi:10.1001/jamanetworkopen.2023.40688

eAppendix 1. Eligibility Survey and Inclusion Criteria

eAppendix 2. Semistructured Interview Guide for Assessing the Perspectives and Experiences of Black Adults With SLE

eFigure. Example of a Conceptual Diagram From the Within-Person Profiles

This supplementary material has been provided by the authors to give readers additional information about their work.

© 2023 Yalavarthi B et al. *JAMA Network Open.*

eAppendix 1. Eligibility survey and inclusion criteria

**Survey questions**	**Inclusion criteria**
What is your age?	Must be 18 years or older
What is your gender?	
What is your race/ethnicity?	Must identify as Black or African American
Do you live in Michigan?	Must be yes
What city in Michigan do you reside in?	
Has a doctor or other health professional ever told you, "You have systemic lupus erythematosus"?	Must be yes
When were you first diagnosed?	
What is the highest degree or level of school you have completed?	
What category describes your current employment status?	
What best describes your current relationship status?	
Including yourself, how many people live in your household?	
How many children under the age of 18 live in your household?	
Do you have access to a phone, computer, tablet, or other device that you can use to participate in the interview?	Must be yes

© 2023 Yalavarthi B et al. *JAMA Network Open.*

eAppendix 2. Semistructured interview guide for assessing the perspectives and experiences of Black adults with SLE[a]

1. Tell me about your experience of being diagnosed with lupus.
2. Next, I'm going to read a list of symptoms that can occur with lupus. Please indicate whether each symptom is something you experience.
3. How do your symptoms affect your day-to-day life?
4. What treatments or strategies help you manage or cope with your symptoms or symptom flares?
5. Tell me about whether doctors or health care providers play a role in managing and coping with lupus.
6. Tell me about whether diet and nutrition play a role in managing and coping with lupus.
7. Tell me about whether physical activity and exercise play a role in managing and coping with lupus.
8. Is there anything else that has been particularly helpful to you as someone living with lupus?
9. What other challenges do you experience as someone living with lupus?
10. How did the pandemic affect your ability to manage your symptoms, if at all?
11. What advice would you give to someone recently diagnosed with lupus?
12. What types of services or resources would you like to see be made available or more available for those living with lupus in your community?
13. What types of studies or research do you think are needed to improve lupus treatment and management?
14. Sometimes research for lupus includes studies that want to collect biological samples, like blood, urine, or skin. The goal of this research is often to inform the development of better treatment strategies. Would you participate in this type of research? Why or why not?
15. Sometimes research for lupus includes studies that assign some volunteers to a treatment group and other volunteers to a placebo or control group. When volunteers are assigned to a study arm, whether the intervention group or the control group, it is usually random, and they may not know what group they are in until the end of the study. The goal of this research is often to test the efficacy of a drug or educational intervention. Would you participate in this type of research? Why or why not?
16. How should researchers improve their communication with participants about the results of a study?

© 2023 Yalavarthi B et al. *JAMA Network Open.*

Next, we have a brief questionnaire to conclude the interview. You can answer yes or no or elaborate if you feel comfortable.

17. I'm going to read a list of activities and strategies that some people use to manage or cope with chronic conditions like lupus. Please indicate whether each strategy is something you use to manage your symptoms.
18. In the past 12 months, has lack of transportation kept you from meetings, work, or getting things needed for daily living?
19. In the last 12 months, did you not see a doctor or skip medications to save money?
20. How often were the following statements true?
    a. Within the past 12 months, you worried that your food would run out before you got money to buy more.
    b. Within the past 12 months, the food you bought just didn't last and you didn't have money to get more.
21. In the last 12 months, has the utility company shut off your service for not paying your bills?
22. In the next 2 months, are you worried that you may not have stable housing?
23. Can you count on anyone to provide you with emotional support such as talking over problems or helping you make a difficult decision?
24. In general, how many close friends do you have? By "close friends" we mean relatives or non-relatives that you feel at ease with, can talk to about private matters, and can call on for help.
25. Lastly, can we contact you with any follow up questions or other lupus-related research opportunities in the future?
26. Is there anything else you would like to add before we end the interview?

---

[a]After piloting the interview guide with 2 participants, we added questions about the pandemic (question 10) and research perceptions (questions 13-16). After 4 interviews, we presented the interview guide to a racially diverse panel of people with lupus and their family members who were not study participants (*n* = 7, aged 31-80 years). Panelists did not suggest additional questions for the guide, but they emphasized the importance of paying attention to family history and the impact of lupus on family relationships during data collection. After 13 interviews, we added a question about what types of resources should be made available or more available for people with SLE (question 12).

© 2023 Yalavarthi B et al. *JAMA Network Open.*

eFigure. Example of a conceptual diagram from the within-person profiles

Conceptual Diagram:

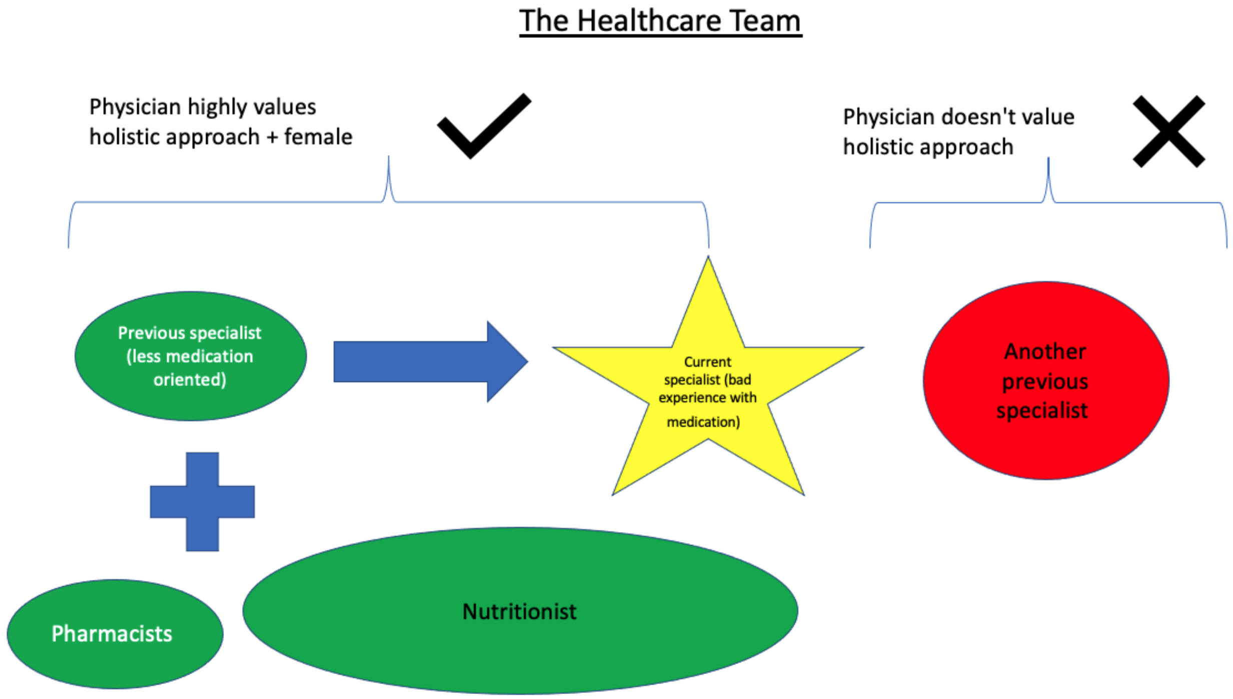

Description: This participant (P02) described both positive and negative aspects of her healthcare team. For P02, having a specialist who prioritized holistic medicine was important. One of P02's past doctors had fit this criterion very well, and P02 described the patient-physician relationship as excellent. This past doctor also recommended a nutritionist who was beneficial to P02 and P02 was still currently working with the nutritionist at the time of the interview (represented by the long oval). P02 did not feel as positive about her current doctor because she had a bad reaction when taking a medication prescribed by the current doctor, which went against P02's holistic approach. P02 also wondered whether she was her current doctor's only African American patient because the doctor said she was the only one who got sick from the medication. However, P02 was still working with this doctor at the time of the interview with the hope of building on their relationship and because P02 felt that this current doctor really listened to her (highlighted through the yellow star). The red circle highlights another doctor from the past who was not encouraging. This previous doctor lacked a holistic approach and was not willing to help P02 achieve her goals. P02 also mentioned that she prefers doctors who are female because she feels more understood by them compared to male doctors. Additionally, P02 described the value of working with a pharmacist who can flag allergies and provide recommendations for adjusting medication dosages.

© 2023 Yalavarthi B et al. *JAMA Network Open.*
