# Supplementary material for: Recommendations for Improving Systemic Lupus Erythematosus Care From Black Adults: A Qualitative Study
Source: JAMA Netw Open. 2023 Oct 31;6(10):e2340688. doi: 10.1001/jamanetworkopen.2023.40688 (PMC10618846; doi:10.1001/jamanetworkopen.2023.40688)
Supplement: Supplement 2. — Data Sharing Statement [file jamanetwopen-e2340688-s002.pdf]

Data Sharing Statement

Yalavarthi. Recommendations for Improving Systemic Lupus Erythematosus Care From Black Adults. *JAMA Netw Open*. Published October 31, 2023.
doi:10.1001/jamanetworkopen.2023.40688

Data
Data available: No
